# Nursing consultations and control of diabetes in general practice: a retrospective observational study

**DOI:** 10.3399/bjgp15X686881

**Published:** 2015-09-28

**Authors:** Trevor Murrells, Jane Ball, Jill Maben, Mark Ashworth, Peter Griffiths

**Affiliations:** National Nursing Research Unit, Florence Nightingale Faculty of Nursing and Midwifery, King’s College London, London.; NIHR CLAHRC Wessex, University of Southampton, Southampton.; National Nursing Research Unit, Florence Nightingale Faculty of Nursing and Midwifery, King’s College London, London.; Department of Primary Care and Public Health Sciences, King’s College London, London.; NIHR CLAHRC Wessex, University of Southampton, Southampton.

**Keywords:** diabetes mellitus, general practice, health workforce, nurses, nursing staff, primary health care

## Abstract

**Background:**

Diabetes affects around 3.6 million people in the UK. Previous research found that general practices employing more nurses delivered better diabetes care, but did not include data on individual patient characteristics or consultations received.

**Aim:**

To examine whether the proportion of consultations with patients with diabetes provided by nurses in GP practices is associated with control of diabetes measured by levels of glycated haemoglobin (HbA1c).

**Design and setting:**

A retrospective observational study using consultation records from 319 649 patients with diabetes from 471 UK general practices from 2002 to 2011.

**Method:**

Hierarchical multilevel models to examine associations between proportion of consultations undertaken by nurses and attaining HbA1c targets over time, controlling for case-mix and practice level factors.

**Results:**

The proportion of consultations with nurses has increased by 20% since 2002 but patients with diabetes made fewer consultations per year in 2011 compared with 2002 (11.6 versus 16.0). Glycaemic control has improved and was more uniformly achieved in 2011 than 2002. Practices in which nurses provide a higher proportion of consultations perform no differently to those where nurse input is lower (lowest versus highest nurse contact tertile odds ratio [OR] [confidence interval {95% CI}]: HbA1c ≤53 mmol/mol (7%) 2002, 1.04 [95% CI = 0.87 to 1.25] and 2011, 0.95 [95% CI = 0.87 to 1.03]; HbA1c ≤86 mmol/mol (10%) 2002, 0.97 [95% CI = 0.73 to 1.29] and 2011, 0.95 [95% CI = 0.86 to 1.04]).

**Conclusion:**

Practices that primarily use GPs to deliver diabetes care could release significant resources with no adverse effect by switching their services towards nurse-led care.

## INTRODUCTION

Diabetes prevalence has increased dramatically over the past 10 years and it is estimated to affect 3.6 million people in the UK^1^ and cost the NHS at least £10 billion a year.^2^ Primary care has become the focal point, with more diabetes care now taking place in GP practices.^3^,^4^ To improve the quality of chronic disease management in primary care, a pay-for-performance scheme, the Quality and Outcomes Framework (QOF), was introduced in 2004–2005 with targets and incentives for improving the quality of care for patients with diabetes.^5^ Much of the work in delivering results against the QOF indicators has been delegated by GPs to nurses.^6^ There was a steady increase in the number of nurses employed in general practice until 2007 when numbers declined, before rising again in 2010 and then remaining comparatively stable thereafter.^7^,^8^ The proportion of consultations undertaken by nurses has increased steadily from 1998 onwards.^9^

Several studies have outlined the changes to practice nurses’ workload and their increased role in caring for those with chronic conditions such as diabetes.^10^–^12^ Models of nurse-led diabetes care have been advocated and positively evaluated in a range of settings including primary care.^13^ Clinical trials have shown that nurses provide comparable high-quality primary care that is complementary to that of their medical colleagues.^14^,^15^ Some have argued that there is considerable scope to further increase the amount of primary care delivered by nurses,^16^,^17^ but the benefits of substitution are disputed.^18^

Evidence is scant of the impact on the quality of diabetes care of a widespread and routine increased nursing contribution. Previous research has used practice level data derived from the QOF to examine several long-term conditions including diabetes, and found that overall, practices with higher levels of practice nurse staffing relative to their list size were associated with improved practice performance.^19^ But findings based on aggregated practice level data are constrained; there is limited ability to risk adjust for individual patient characteristics.

This study aimed to examine whether different patterns of workforce activity in primary care are associated with variation in the level of diabetes control as measured by glycated haemoglobin (HbA1c). Specifically, the extent to which patients with diabetes had consultations with registered nurses at the practice level, as opposed to GPs, was examined, and whether there was any association between this and achieving glycaemic control thresholds.

**How this fits in**

The relationship between nurse staffing and patient outcomes has been extensively explored in acute hospital care, but little research has been undertaken in primary care. A previous study used aggregated practice data and found that general practices employing more practice nurses delivered better care for patients with diabetes. This study goes further by including data on individual patient characteristics and on the consultations undertaken by registered nurses, doctors, and other staff. This study shows that there may be considerable scope for increasing the amount of diabetes care that is delivered by nurses.

## METHOD

A retrospective observational design using routinely collected data to examine the associations between staffing inputs at the practice level and glycaemic control at the patient level.

### Data source

Data were derived from electronic patient records from the 556 general practices contributing to The Health Information Network (THIN) database. At the time of the study the database covered 3.7 million active patients in the UK (6% of all patients). THIN has a similar demographic profile and diabetes prevalence to the UK population, although THIN has more patients from affluent areas and fewer younger people.^20^

### Selection of patients with diabetes and GP practices from THIN

Patients registered with a THIN GP practice anytime up to 16 May 2012 with a diabetes related entry in their medical record or health details were initially selected (*n* = 406 632). Diabetes was clearly indicated for 319 649 (79%) of those patients based on a combination of the following: presence of a diagnosis, annual review, HbA1c reading ≥6.5%, and diabetes therapies. A review of a sub-sample of the 86 551 (21%) patients excluded suggested that the selection had been successfully applied.

The selection criteria for GP practices were then applied to the dataset. For each year practices had to meet the following criteria:

- complete mortality data;
- at least 90% of all HbA1c measurements recorded in, or transformable into, percentage units; and
- at least 90% of consultations could be associated with a staff member with known clinical role (not administrative staff).

The number of GP practices selected increased from 2002 to 2009 and then declined (Table 1). The number of patients in the dataset with diabetes increased from 51 493 in 2002 to 150 023 in 2011 (Table 1).

**Table 1.** Population achievement for HbA1c by threshold and level of nurse involvement

**Nurse contact, %**	**2002**	**2003**	**2004**	**2005**	**2006**	**2007**	**2008**	**2009**	**2010**	**2011**
**HbA1c threshold ≤53 mmol/mol (7%)**										
Low: <26.0	30.8	33.9	37.5	37.7	42.2	41.8	43.6	42.9	43.0	42.5
Medium: 26.0–35.3	30.3	34.6	37.3	38.6	41.2	42.6	43.1	43.9	43.7	42.0
High: ≥35.4	31.0	36.6	38.1	40.3	43.1	42.8	42.8	43.6	43.8	43.4

**HbA1c threshold ≤86 mmol/mol (10%)**										
Low: <26.0	71.6	76.9	81.7	83.2	83.2	83.1	84.0	82.8	82.8	82.0
Medium: 26.0–35.3	70.4	77.5	82.8	83.6	83.2	83.5	84.0	83.7	83.3	82.8
High: ≥35.4	74.1	80.4	83.5	84.5	84.8	83.7	83.3	83.0	83.6	82.9

Consultations with nurses, %	30.7	31.0	30.7	30.9	30.6	31.2	31.2	31.5	31.1	32.2
Patients, *n*	51 493	78 502	86 838	103 391	114 039	123 504	135 420	143 329	148 221	150 023
GP practices, *n*	247	375	386	427	441	448	470	471	466	445

### Measures

#### Workforce input — staff consultations

Consultations were defined as direct contact with patients (for example, surgery, clinic, home visit, or telephone conversations). The staff group of the member that made an entry for the consultation was obtained from the medical, health details, and medication records (practice-prescribed therapies). Each consultation was attached to a staff group code (for example, doctor only, doctor, and practice nurse).

Three measures of workforce activity were derived:

- mean number of times patients with diabetes were seen by a healthcare professional (per annum);
- percentage of consultations annually involving practice nurses; or
- total time spent in consultations (minutes) annually and mean consultation length.

#### Outcome glycaemic control

The proportion of patients with diabetes in each practice achieving a certain level of glycaemic control (measured using Hb1Ac) was the main outcome measure (‘population attainment’). The HbA1c reading closest to 1 July was selected for each person with diabetes for each calendar year from 2002 to 2011. Each percentage HbA1c reading was then categorised according to whether it met a lower (≤53 mmol/mol [7%]) or upper (≤86 mmol/mol [10%]) threshold. These lower and upper limits span the range of thresholds used since QOF was introduced in 2004. The 53 mmol/mol (7%) threshold was used in 2009–2010 and 2010–2011, and the 86 mmol/mol (10%) threshold from 2004–2005 to 2008–2009.^21^,^22^

### Statistical analysis

A multilevel modelling approach^23^ was used to test the association between a person with diabetes meeting the HbA1c threshold and the percentage of practice consultations with a nurse. Because the amount of contact is largely determined by clinical need, associations between outcomes were analysed at the patient level and workforce inputs at the practice level were analysed. Individual consultations are likely to be driven primarily by patient factors, whereas activity at the practice level is more reflective of a practice’s approach to care delivery, once patient characteristics are taken into account.

As the underlying population has changed because of earlier diagnosis and treatment, data were analysed by year, rather than longitudinally. A two-level random intercepts hierarchical logistic regression model was fitted with patients nested within practice.

Each model included the following variables:

- patient level: age, sex, ethnic group, case severity (primary care equivalent of the Charlson Index),^24^ obesity, and social deprivation^25^ (Townsend);
- practice level: list size, diabetes prevalence, and UK country; and
- staff activity: percentage of consultations with a nurse and mean number of consultations with a healthcare professional annually.

The following variables were used in the model in their standardised form (mean zero, standard deviation of one): age, case severity, practice list size, prevalence, and consultations. For each year, models were fitted to the lower (≤53 mmol/mol [7%]) and upper (≤86 mmol/mol [10%]) HbA1c threshold using SAS GLIMMIX.

## RESULTS

**Figure 1.**
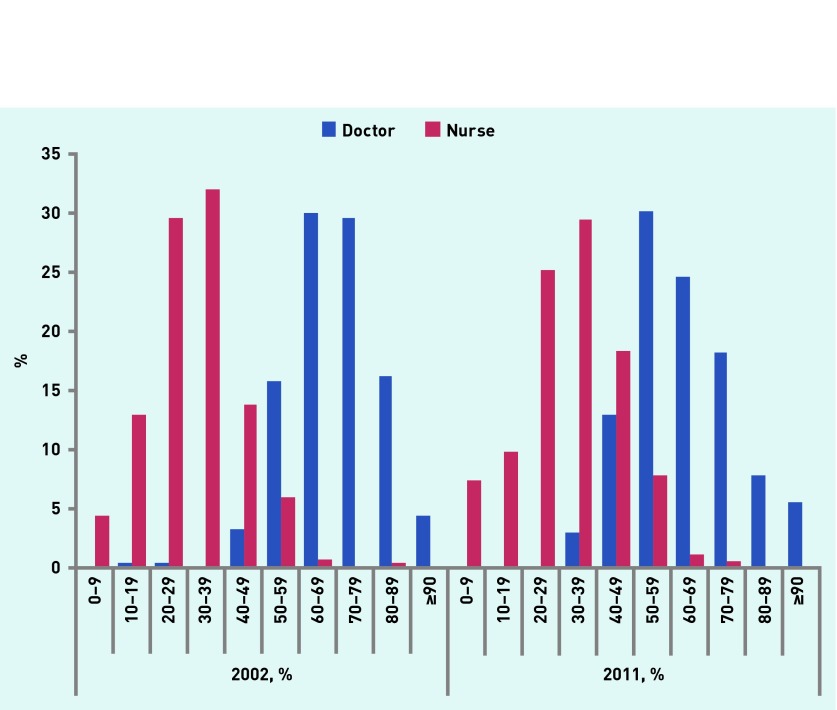
***Proportion of consultations undertaken by doctors and nurses at the practice level in 2002 and 2011. x-axis: proportion of consultations undertaken within each year grouped into 10% bands.***

The mean number of patients with diabetes in each practice increased from 3.0% of all patients registered in 2002 to 4.8% in 2011, and the number with at least one other comorbidity increased from 79.5% in 2002 to 88.2% in 2011. The total annual number of consultations in each practice with patients that have diabetes increased by 12% from 3900 in 2002 to 4376 in 2011. Nurses increased their activity much more than doctors during this period: a 20% increase compared with no change for GPs. In 2002 70% of consultations were undertaken by doctors, falling to 64% in 2011. Meanwhile the mean proportion of consultations undertaken by nurses in each practice increased slightly (from 31% to 32%) (Table 1) and those by other healthcare professionals from <3% to 8%. Variation between practices in the use of nurses also increased. In 2002 for practices in the bottom 10% of nurse activity, ≤15% contacts involved a nurse, whereas in the top 10% this rose to >46%. For 2011 it was 15% and 50%, respectively. These changes are explored in more detail in Figure 1.

Activity levels have not increased as sharply as the number of patients with diabetes, so although both doctors and nurses are providing more consultations, each patient with diabetes received fewer consultations in 2011 (11.6) than in 2002 (16.0). The mean duration of a consultation with a doctor increased from 10.2 in 2002 to 11.1 minutes in 2011, and for nurses from 11.2 to 12.8 minutes.

The mean number of times patients had their diabetes reviewed fell from 1.23 in 2002 to 1.05 in 2007 before rising again to 1.31 in 2011. The corresponding means for reviews by doctors were 0.48, 0.31, and 0.34, and for nurses 0.72, 0.71, and 0.90. The net result was an increase in the proportion of diabetes reviews undertaken by nurses, from 58% in 2002 to 69% in 2011.

**Figure 2.**
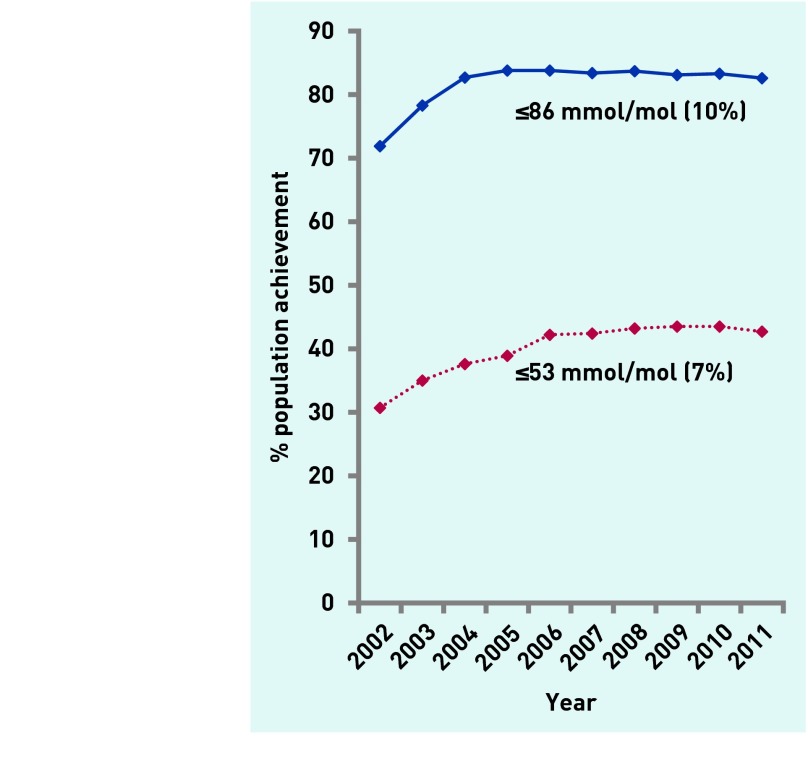
***HbA1c population achievement by threshold.***

Glycaemic control improved considerably between 2002 and 2004. From then on improvement slowed, reaching a plateau in 2005 for the upper threshold (≤86 mmol/mol [10%]) and in 2009 for the lower threshold (≤53 mmol/mol [7%]) (Figure 2). There was more variation between practices, after adjustment, in 2002 than in subsequent years (2003–2011) for both the lower and higher thresholds (Residual σ^2^, Tables 2 and 3).

**Table 2.** Multilevel model examining level of nurse contact and meeting the HbA1c ≤53 mmol/mol (7%) threshold

**Year**	**Nurse contact, OR (95% CI)**			**Consultations per healthcare professional, OR (95% CI)**	**Residual σ^2^**

	**Low**	**Medium**	**High**		
2002	1.04 (0.87 to 1.25)	0.94 (0.79 to 1.11)	1.00 (−)	0.90^a^ (0.82 to 0.98)	0.53
2003	0.86 (0.77 to 0.95)	0.89 (0.80 to 1.00)	1.00 (−)^a^	0.95^a^ (0.91 to 1.00)	0.39
2004	0.95 (0.86 to 1.06)	0.96 (0.87 to 1.07)	1.00 (−)	0.97 (0.93 to 1.01)	0.38
2005	0.90 (0.82 to 0.99)	0.91 (0.83 to 1.07)	1.00 (−)	0.97 (0.93 to 1.00)	0.36
2006	0.97 (0.88 to 1.06)	0.93 (0.85 to 1.02)	1.00 (−)	0.98 (0.94 to 1.02)	0.36
2007	0.94 (0.85 to 1.03)	0.98 (0.90 to 1.08)	1.00 (−)	1.01 (0.96 to 1.05)	0.38
2008	1.01 (0.92 to 1.10)	1.01 (0.93 to 1.10)	1.00 (−)	1.02 (0.99 to 1.05)	0.36
2009	0.94 (0.86 to 1.03)	1.01 (0.93 to 1.11)	1.00 (−)	1.02 (0.99 to 1.05)	0.38
2010	0.96 (0.88 to 1.04)	0.98 (0.91 to 1.07)	1.00 (−)	1.03 (1.00 to 1.07)	0.35
2011	0.95 (0.87 to 1.03)	0.95 (0.88 to 1.04)	1.00 (−)	1.04^a^ (1.01 to 1.08)	0.34

Nurse contact: probability >χ^2^ (with 2 degrees of freedom). Consultations per healthcare professional: probability > t.

a P<0.05.

**Table 3.** Multilevel model examining level of nurse contact and meeting the HbA1c ≤ 86 mmol/mol (10%) threshold

**Year**	**Nurse contact, OR (95% CI)**			**Consultations per healthcare professional, OR (95% CI)**	**Residual σ^2^**

	**Low**	**Medium**	**High**		
2002	0.97 (0.73 to 1.29)	0.83 (0.63 to 1.09)	1.00 (−)	0.83^b^ (0.72 to 0.95)	0.87
2003	0.83 (0.72 to 0.95)	0.84 (0.73 to 0.97)	1.00 (−)^a^	0.90^c^ (0.85 to 0.95)	0.52
2004	0.91 (0.81 to 1.02)	0.97 (0.86 to 1.09)	1.00 (−)	0.96 (0.91 to 1.01)	0.42
2005	0.93 (0.84 to 1.03)	0.95 (0.86 to 1.05)	1.00 (−)	0.98 (0.93 to 1.02)	0.38
2006	0.92 (0.84 to 1.03)	0.92 (0.83 to 1.02)	1.00 (−)	0.99 (0.94 to 1.03)	0.41
2007	0.99 (0.89 to 1.10)	1.02 (0.92 to 1.13)	1.00 (−)	0.99 (0.95 to 1.04)	0.41
2008	1.04 (0.95 to 1.15)	1.08 (0.98 to 1.19)	1.00 (−)	1.00 (0.97 to 1.03)	0.39
2009	1.00 (0.91 to 1.10)	1.07 (0.97 to 1.18)	1.00 (−)	0.99 (0.96 to 1.02)	0.40
2010	0.98 (0.89 to 1.07)	1.02 (0.92 to 1.11)	1.00 (−)	0.99 (0.96 to 1.03)	0.37
2011	0.95 (0.86 to 1.04)	0.99 (0.90 to 1.09)	1.00 (−)	1.01 (0.96 to 1.05)	0.38

Nurse contact: probability >χ^2^ (with 2 degrees of freedom). Consultations per healthcare professional: probability > t.

a P<0.05.

b P<0.01.

c P<0.001.

The proportion of nurse contact at the practice level was categorised into three groups of roughly equal size: low (<26.0%), medium (26.0–35.3%), and high (≥35.4%), using 2002 as the reference year. The proportion of patients attaining the ‘tight’ (HbA1c ≤53 mmol/mol [7%]) threshold was consistently higher in practices with a high proportion of nurse contact for every year from 2002 to 2007. In absolute terms, however, the differences were generally small. The difference between the high and low contact groups for the higher threshold (HbA1c ≤86 mmol/mol [10%]) was more apparent in the earlier period with maximum advantage of 3.5% (2003) and consistently in excess of 1% before 2007 (Table 1).

After risk adjustment at the person and practice level, practices in which patients had a higher proportion of nurse contact had significantly more patients meeting both the lower (≤53 mmol/mol [7%]) and higher threshold (≤86 mmol/mol [10%]) in 2003 (Tables 2 and 3). For all other years the proportion of patients meeting either threshold did not vary significantly across nurse contact groups.

Practices with a higher mean annual number of consultations with a healthcare professional had significantly fewer patients meeting both the lower and higher thresholds for 2002 and 2003 (Tables 2 and 3). This association was stronger for the higher threshold (2002: *P* = 0.007, 2003: *P*<0.001) than the lower threshold (2002: *P* = 0.015, 2003: *P* = 0.047). For the lower threshold the association has changed, with higher numbers of consultations reducing the chances of the threshold being met in the first half of the period (2002–2006) and increasing the chances of the threshold being met in the second half (2007–2011). This association was statistically significant in 2011 (*P* = 0.020) and close to significance in 2010 (*P* = 0.066).

## DISCUSSION

### Summary

Over the past 10 years, glycaemic control has improved in patients of UK general practices and is now more uniformly achieved across practices, despite a large increase in recorded prevalence of diabetes. Much of the variation between practices in the population achieving glycaemic control is related to differences between the patient populations served.

As diabetes has become increasingly prevalent, proportionally more care is being delivered and managed by nurses, although the changes have not been as substantial as might have been supposed. The amount of diabetes care delivered by nurses varies substantially between practices from ≤15% of all contacts in the bottom 10% of practices, in terms of nurse activity, to almost a half of all contacts in the top 10%. Patients with diabetes now have fewer consultations per year than in the past, but more of them are undertaken by nurses who provide slightly longer consultations than GPs. Practices in which nurses undertake a higher proportion of consultations with patients with diabetes perform no differently to those in which nurse input is lower.

In the early part of the decade practices undertaking higher numbers of consultations were less likely to meet the QOF thresholds, suggesting practices may have been ‘playing catch-up’. Practices where patients were already meeting QOF thresholds could maintain those levels without increasing their work activity. From the middle of the decade onwards the usual expectation of higher level of work activity and meeting the thresholds took hold.

### Strengths and limitations

This study analysed individual patient data from practices that care for 6% of the UK population. Prevalence and consultation rates in this study are similar to those found elsewhere,^1^,^7^,^26^ and this provides some validation of the calculations and computations made. A ‘consultation’ could not be definitively assigned to one member of staff if more than one had made changes to the record(s) associated with that consultation, nor could time be apportioned to individual staff groups if that was the case.

All types of consultation were used to reflect the total care provided by a practice to patients with diabetes. Focusing on consultations with a diabetes Read Code would have resulted in a high level of data attrition. Care of related complications (for example, hypertension) would have been missed particularly where GPs only code a single comorbidity because of time constraints.

The accuracy of consultation time is unclear as it relies on practitioners remembering to mark the patient as ‘left’. The use of electronic systems was well established in all practices, however there is no reason to assume that errors varied systematically across years.

A single measure such as HbA1c may not provide an absolute indication of whether a person’s diabetes is well controlled or not; meeting HbA1c thresholds was chosen as it has been one of the key QOF diabetes indicators. The cross-sectional analysis did not allow for testing for causality, but, nonetheless, the findings establish that practices that involve nurses more often typically perform as well as those that use nurses less often (in terms of glycaemic control). Practices vary in their availability of resources, for example patient list per nurse and doctor, and the availability of diabetes specialist GP or nurses. It was not possible to consider these practice level contextual factors in the present analysis, although a sub-study based on a survey of 249 practices found no associations between specialist provision and HbA1c level.^27^ The richness of the GP patient level data in this study is mirrored by a paucity of good-quality workforce data.^8^

### Comparison with existing literature

Specialist nurses or nurse case managers have been shown to be associated with improved short-term diabetic control in a number of trials,^28^ and trials have demonstrated that nurse practitioners can achieve outcomes that are equivalent to those of GPs.^29^ However, little research has examined the impact of the increased use of nurses in routine general practice outside of these trials. Associations were found previously between higher levels of nurse staffing and better performance measured using QOF diabetes domain score and per cent meeting the lower (≤57 mmol/mol [7%]) and upper (≤86 mmol/mol [10%]) HbA1c QOF indicator thresholds for 2005–2006^19^,^30^ using aggregated practice level data only, with no control for individual patient characteristics. The present findings based on 2005 THIN data are consistent with this earlier work. This study provides support for the view that extension of the nursing role would not compromise quality of care or patient outcomes.^31^

### Implications for research and practice

This study has important implications for policy, practice, and further research. Nurses are well placed to help patients with diabetes make the behavioural changes that can benefit their long-term health. This study shows that there may be considerable scope for increasing the amount of diabetes care that is delivered by nurses, although this would have workforce implications. Some practices may have to employ more nurses to increase capacity to meet the demand. For others, the amount of care delivered by nurses is still low.

Further evidence is required concerning the long-term costs and benefits of deploying more nurses to undertake diabetes care but the results of this study give no indication that the control of diabetes is worse when more care is provided by nurses. Future research should also consider the preferences of patients with diabetes and a full economic analysis.
